# Supplementary material for: A systematic review and meta-analysis of unimodal and multimodal predation risk assessment in birds
Source: Nat Commun. 2024 May 18;15:4240. doi: 10.1038/s41467-024-48702-6 (PMC11102462; doi:10.1038/s41467-024-48702-6)
Supplement: Supplementary file 1 — Supplementary Information [file 41467_2024_48702_MOESM1_ESM.pdf]

Supplementary Information for: A systematic review and meta-analysis of unimodal and multimodal predation risk assessment in birds

Kimberley J. Mathot[1,2], Josue David Arteaga-Torres[1], Anne Besson[1,3], Deborah M. Hawkshaw[1], Natasha Klappstein[1,4], Rebekah A. McKinnon[1], Sheeraja Sridharan[1], and Shinichi Nakagawa[5, 6]

1. Department of Biological Sciences, University of Alberta, Edmonton, Alberta, Canada

2. Canada Research Chair in Integrative Ecology, Department of Biological Sciences, University of Alberta

3. Current address: Department of Zoology, University of Otago, New Zealand

4. Current address: Department of Statistics, Dalhousie University, Halifax, Nova Scotia, Canada

5. Evolution & Ecology Research Centre and School of Biological, Earth and Environmental Sciences, University of New South Wales, Sydney NSW 2052, Australia

6. Theoretical Sciences Visiting Program, Okinawa Institute of Science and Technology Graduate University, Onna, 904-0495, Japan

Figure S1: PRISMA flow chart showing the number of articles discovered and/or retained at each phase of the systematic review. All studies included in the meta-analysis are indicated with asterisks in the References section of the main text, and the list of all studies that were rejected after reading the full text can be found in Supplementary Information Table S3 including the reason for the rejection............ 2

Table S1. Descriptions of trait categories used for coding response variables. When the variable was scored in a way that was inverse to the investment in the trait it measured, the direction of the estimate was corrected by multiplying by -1 (indicated in parentheses following the example description as applicable). For example, inter-scan-interval is inversely related to investment in vigilance, and was therefore multiplied by -1. The predicted effect of an increased perceived predation risk on a category of response variable is indicated in the column “Predicted effect”. In order to allow to estimate global effects in our meta-analyses, all variables were coded so that effects in the predicted direction were positive. This means that estimates for categories where the predicted effect was “Decrease” were all multiplied by -1 prior to analysis. 3

Table S2. PRISMA Eco-Evo reporting checklist based on O'Dea, et al. [1]. 6

Table S3: List of studies that were excluded from the meta-analysis based on reading of full text, and their reasons for exclusion.. 12

Table S4: List of variables extracted from articles included in the meta-analysis and rationale. 14

Supplementary References.. 15

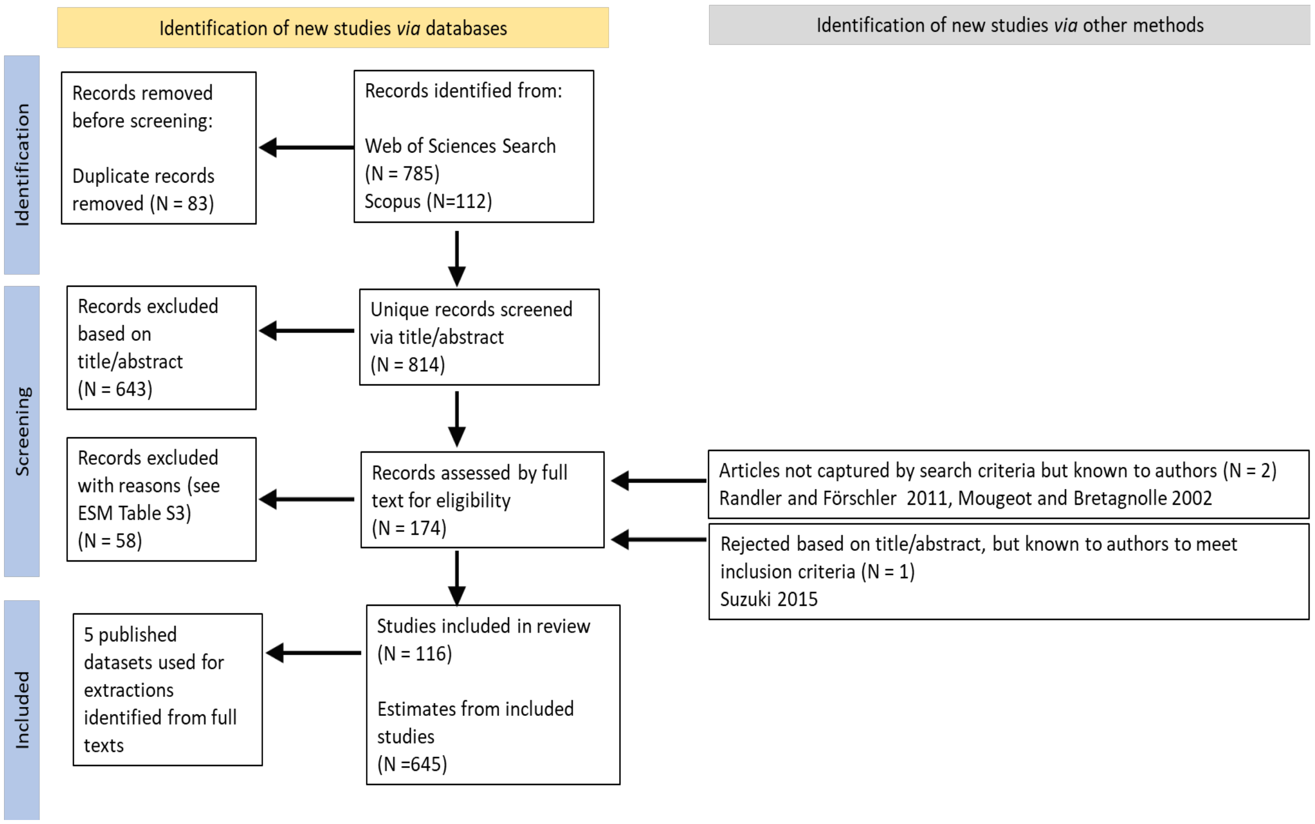

Figure S1: PRISMA flow chart showing the number of articles discovered and/or retained at each phase of the systematic review. All studies included in the meta-analysis are indicated with asterisks in the References section of the main text, and the list of all studies that were rejected after reading the full text can be found in Supplementary Information Table S3 including the reason for the rejection.

Table S1. Descriptions of trait categories used for coding response variables. When the variable was scored in a way that was inverse to the investment in the trait it measured, the direction of the estimate was corrected by multiplying by -1 (indicated in parentheses following the example description as applicable). For example, inter-scan-interval is inversely related to investment in vigilance, and was therefore multiplied by -1. The predicted effect of an increased perceived predation risk on a category of response variable is indicated in the column “Predicted effect”. In order to allow to estimate global effects in our meta-analyses, all variables were coded so that effects in the predicted direction were positive. This means that estimates for categories where the predicted effect was “Decrease” were all multiplied by -1 prior to analysis.

**Type**	**Category (*definition*)**	**Subcategories**	**Predicted Effect**	**Examples**
Behaviour	Antipredator (*Behaviours that mitigate predation risk by increasing likelihood of detecting predator, increasing likelihood of avoiding predator, or decreasing likelihood that predator can mount a successful attack)*	Vigilance<br>Avoidance<br>Mobbing/ alarming	Increase	Scanning rate<br>Inter-scan interval (-1)<br>Mean scan bout duration<br>Inspection behaviour<br>Choice of non-predator location<br>Choice of predator location (-1)<br>Use of cover<br>Avoidance of cover (-1)<br>Dispersal<br>Escape flight behaviour<br>Settlement (density of breeding pairs) (-1)<br>Settlement timing<br>Probability of building a nest (-1)<br>Nest abandonment<br>Time out of refuge (-1)<br>Freezing behaviour<br>Probability of approaching to speaker/mount<br>Call rate<br>Wing flicking<br>Recruitment of conspecifics
	Costly Behaviours (*Behaviours that expend net energy but that are not involved in mitigating predation risk*)	Courtship<br>Activity	Decrease	Number of songs<br>Songs per bird<br>Count of mate attraction calls<br>Duration of courtship display<br>Singing rate<br>Exploration<br>Number of movements<br>Latency to begin activity (-1)<br>Proportion of time active
	Intake (*Behaviours that bring in net energy*	Foraging<br>Begging	Decrease	Foraging rates<br>Latency to resume feeding (-1)<br>Giving up density (-1)

	but that are not involved in mitigating predation risk)			Probability of returning to feeder (-1)<br>Begging per hour<br>Begging rate<br>Gapes per sec
	Parental Care (*Care provided to eggs or offspring*)	Incubation/ brooding<br>Provisioning	Decrease	Number of incubation bouts<br>Mean incubation bout duration<br>Proportion of time incubating<br>Probability of abandoning nest (-1)<br>Number of brooding bouts<br>Total time brooding<br>Number/rate of incubation feeds<br>Provisioning rate<br>Provisioning biomass delivery<br>Provisioning load size<br>Latency to resume provisioning (-1)
Life History	Reproduction (*Measures of reproductive success*)	ClutchSize<br>EggSize<br>Nestlings	Decrease	Clutch size<br>Mean clutch size<br>Clutch size change across years<br>Egg mass<br>Egg volume<br>Brood size<br>Number of nestlings<br>Number of fledglings<br>Hatching Success<br>Prop chicks dead (-1)<br>Prop eggs not hatched (-1)<br>Clutch mass<br>Fledging success<br>Brood mass<br>Hatching success
	Phenology (*Measures of timing of key life history events*)	LayDate<br>Developmental timing	Increase	Lay date<br>Clutch initiation date<br>Nest initiation date<br>Duration of incubation<br>Duration of nestling<br>Age at fledging<br>Rate of ageing (telomere length)<br>Time to hatching
Physiology	Condition (*Measure of body condition of individuals*)	Mass<br>Growth	Decrease	Morning mass<br>Average mass<br>Evening mass<br>Rate of mass gain (adults)<br>Lean mass<br>Fat mass<br>Body mass<br>Residual fat

				Asymptotic body mass (nestlings)<br>Mass gain (nestlings)<br>Sing growth rate<br>Tarsus growth rate<br>Structural body size
	Hormones (*Measure of hormone levels in vivo)*	Corticosterone	Increase	Corticosterone<br>Basal corticosterone<br>Stress corticosterone<br>Maximum corticosterone level

Table S2. PRISMA Eco-Evo reporting checklist based on O'Dea, et al. [1].

Checklist item	Sub-item number	Sub-item	Reported by authors?	Notes
Title and abstract	1.1	Identify the review as a systematic review, meta-analysis, or both	Yes	
	1.2	Summarise the aims and scope of the review	Yes	
	1.3	Describe the data set	Yes	
	1.4	State the results of the primary outcome	Yes	
	1.5	State conclusions	Yes	
	1.6	State limitations	Yes	
Aims and questions	2.1	Provide a rationale for the review	Yes	
	2.2	Reference any previous reviews or meta-analyses on the topic	Yes	
	2.3	State the aims and scope of the review (including its generality)	Yes	
	2.4	State the primary questions the review addresses (e.g. which moderators were tested)	Yes	
	2.5	Describe whether effect sizes were derived from experimental and/or observational comparisons	Yes	Details provided in the methods section
Review registration	3.1	Register review aims, hypotheses (if applicable), and methods in a time-stamped and publicly accessible archive and provide a link to the registration in the methods section of the manuscript. Ideally registration occurs before the search, but it can be done at any stage before data analysis.	No	
	3.2	Describe deviations from the registered aims and methods	No	
	3.3	Justify deviations from the registered aims and methods	No	
Eligibility criteria	4.1	Report the specific criteria used for including or excluding studies when screening titles and/or abstracts, and full texts, according to the aims of the systematic review (e.g. study design, taxa, data availability)	Yes	
	4.2	Justify criteria, if necessary (i.e. not obvious from aims and scope)	Yes	
Finding studies	5.1	Define the type of search (e.g. comprehensive search, representative sample)	Yes	

	5.2	State what sources of information were sought (e.g. published and unpublished studies, personal communications)	Yes	
	5.3	Include, for each database searched, the exact search strings used, with keyword combinations and Boolean operators	Yes	
	5.4	Provide enough information to repeat the equivalent search (if possible), including the timespan covered (start and end dates)	Yes	
Study selection	6.1	Describe how studies were selected for inclusion at each stage of the screening process (e.g. use of decision trees, screening software)	Yes	
	6.2	Report the number of people involved and how they contributed (e.g. independent parallel screening)	Yes	
Data collection process	7.1	Describe where in the reports data were collected from (e.g. text or figures)	Yes	
	7.2	Describe how data were collected (e.g. software used to digitize figures, external data sources)	Yes	
	7.3	Describe moderator variables that were constructed from collected data (e.g. number of generations calculated from years and average generation time)	Yes	Treatment duration calculated in days. Where duration < 1 day, proportion of day calculated assuming 12hr daylength
	7.4	Report how missing or ambiguous information was dealt with during data collection (e.g. authors of original studies were contacted for missing descriptive statistics, and/or effect sizes were calculated from test statistics)	Yes	
	7.5	Report who collected data	Yes	
	7.6	State the number of extractions that were checked for accuracy by co-authors	Yes	
Data items	8.1	Describe the key data sought from each study	Yes	
	8.2	Describe items that do not appear in the main results, or which could not be extracted due to insufficient information	Yes	
	8.3	Describe main assumptions or simplifications that were made (e.g. categorising both 'length' and 'mass' as 'morphology')	Yes	

	8.4	Describe the type of replication unit (e.g. individuals, broods, study sites)	Yes	
Assessment of individual study quality	9.1	Describe whether the quality of studies included in the systematic review or meta-analysis was assessed (e.g. blinded data collection, reporting quality, experimental *vs*. observational)	No	All studies included were experimental
	9.2	Describe how information about study quality was incorporated into analyses (e.g. meta-regression and/or sensitivity analysis)	No	Information on quality was not incorporated into analyses
Effect size measures	10.1	Describe effect size(s) used	Yes	
	10.2	Provide a reference to the equation of each calculated effect size (e.g. standardised mean difference, log response ratio) and (if applicable) its sampling variance	Yes	
	10.3	If no reference exists, derive the equations for each effect size and state the assumed sampling distribution(s)	NA	A reference for the effect size and its sampling variance was available, so no derivation was required
Missing data	11.1	Describe any steps taken to deal with missing data during analysis (e.g. imputation, complete case, subset analysis)	NA	There were no missing data
	11.2	Justify the decisions made to deal with missing data	NA	There were no missing data
Meta-analytic model description	12.1	Describe the models used for synthesis of effect sizes	Yes	
	12.2	The most common approach in ecology & evolution will be a random-effects model, often with a hierarchical/multilevel structure. If other types of models are chosen (e.g. common/fixed effects model, unweighted model), provide justification for this choice	Yes	
Software	13.1	Describe the statistical platform used for inference (e.g. *R*)	Yes	
	13.2	Describe the packages used to run models	Yes	
	13.3	Describe the functions used to run models	Yes	
	13.4	Describe any arguments that differed from the default settings	Yes	
	13.5	Describe the version numbers of all software used	Yes	
Non-independence	14.1	Describe the types of non-independence encountered (e.g. phylogenetic, spatial, multiple measurements over time)	Yes	

	14.2	Describe how non-independence has been handled	Yes
	14.3	Justify decisions made	Yes
Meta-regression and model selection	15.1	Provide a rationale for the inclusion of moderators (covariates) that were evaluated in meta-regression models	Yes
	15.2	Justify the number of parameters estimated in models, in relation to the number of effect sizes and studies (e.g. interaction terms were not included due to insufficient sample sizes)	Yes
	15.3	Describe any process of model selection	Yes
Publication bias and sensitivity analyses	16.1	Describe assessments of the risk of bias due to missing results (e.g. publication, time-lag, and taxonomic biases)	Yes
	16.2	Describe any steps taken to investigate the effects of such biases (if present)	Yes
	16.3	Describe any other analyses of robustness of the results, e.g. due to effect size choice, weighting or analytical model assumptions, inclusion or exclusion of subsets of the data, or the inclusion of alternative moderator variables in meta-regressions	Yes
Clarification of *post hoc* analyses	17.1	When hypotheses were formulated after data analysis, this should be acknowledged.	Yes
Metadata, data, and code	18.1	Share metadata (i.e. data descriptions)	Yes
	18.2	Share data required to reproduce the results presented in the manuscript	Yes
	18.3	Share additional data, including information that was not presented in the manuscript (e.g. raw data used to calculate effect sizes, descriptions of where data were located in papers)	Yes
	18.4	Share analysis scripts (or, if a software package with graphical user interface (GUI) was used, then describe full model specification and fully specify choices)	Yes
Results of study selection process	19.1	Report the number of studies screened	Yes
	19.2	Report the number of studies excluded at each stage of screening	Yes
	19.3	Report brief reasons for exclusion from the full-text stage	Yes
	19.4	Present a Preferred Reporting Items for Systematic Reviews and Meta-Analyses (PRISMA)-like flowchart (www.prisma-statement.org).	Yes

Sample sizes and study characteristics	20.1	Report the number of studies and effect sizes for data included in meta-analyses	Yes	
	20.2	Report the number of studies and effect sizes for subsets of data included in meta-regressions	Yes	
	20.3	Provide a summary of key characteristics for reported outcomes (either in text or figures; e.g. one quarter of effect sizes reported for vertebrates and the rest invertebrates)	Yes	
	20.4	Provide a summary of limitations of included moderators (e.g. collinearity and overlap between moderators)	Yes	
	20.5	Provide a summary of characteristics related to individual study quality (risk of bias)	NA	The quality of studies included in the meta-analysis was not assessed
Meta-analysis	21.1	Provide a quantitative synthesis of results across studies, including estimates for the mean effect size, with confidence/credible intervals	Yes	
Heterogeneity	22.1	Report indicators of heterogeneity in the estimated effect (e.g. I^2, tau^2 and other variance components)	Yes	
Meta-regression	23.1	Provide estimates of meta-regression slopes (i.e. regression coefficients) and confidence/credible intervals	Yes	
	23.2	Include estimates and confidence/credible intervals for all moderator variables that were assessed (i.e. complete reporting)	Yes	
	23.3	Report interactions, if they were included	NA	No interactions were included
	23.4	Describe outcomes from model selection, if done (e.g. R^2 and AIC)	Yes	
Outcomes of publication bias & sensitivity analyses	24.1	Provide results for the assessments of the risks of bias (e.g. Egger's regression, funnel plots)	Yes	
	24.2	Provide results for the robustness of the review's results (e.g. subgroup analyses, meta-regression of study quality, results from alternative methods of analysis, and temporal trends)	Yes	
Discussion	25.1	Summarise the main findings in terms of the magnitude of effect	Yes	
	25.2	Summarise the main findings in terms of the precision of effects (e.g. size of confidence intervals, statistical significance)	Yes	

	25.3	Summarise the main findings in terms of their heterogeneity	Yes	
	25.4	Summarise the main findings in terms of their biological/practical relevance	Yes	
	25.5	Compare results with previous reviews on the topic, if available	Yes	
	25.6	Consider limitations and their influence on the generality of conclusions, such as gaps in the available evidence (e.g. taxonomic and geographical research biases)	Yes	
Contributions and funding	26.1	Provide names, affiliations, and funding sources of all co-authors	Yes	
	26.2	List the contributions of each co-author	Yes	
	26.3	Provide contact details for the corresponding author	Yes	
	26.4	Disclose any conflicts of interest	NA	There were no conflicts of interest
References	27.1	Provide a reference list of all studies included in the systematic review or meta-analysis	Yes	
	27.2	List included studies as referenced sources (e.g. rather than listing them in a table or supplement)	Yes	

Table S3: List of studies that were excluded from the meta-analysis based on reading of full text, and their reasons for exclusion.

Reference	Reason for Exclusion
Amo, et al. [2]	Predator treatment is novel to focal species
Amo, et al. [3]	Predator treatment is novel to focal species
Andreasson, et al. [4]	Response variable (Tb) not replicated in other studies
Antze and Koper [5]	Mobbing calls used were generic response to humans
Atkins, et al. [6]	Doesn't meet minimum sample size requirements. Level of analysis is "site", with N = 1 treatment and N = 2 control.
Aviles, et al. [7]	Mixed species level data
Berziņš, et al. [8]	No control treatment
Blackwell, et al. [9]	Results do not allow for extraction of effects for each level of treatment separately
Breviglieri and Romero [10]	Mixed species level data.
Cimprich, et al. [11]	Season (migration) had insufficient independent studies for inclusion (K = 2)
Coslovsky and Richner [12]	Not eligible – study of transgenerational effect of predator treatments. Traits investigated in nestlings that were not exposed to cues of predation directly.
da Cunha, et al. [13]	Mixed species level data
Davies and Welbergen [14]	Mixed species level data
Dutour, et al. [15]	Mixed species level data
Fardell, et al. [16]	Mixed species level data
Forsman and Monkkonen [17]	Mixed species level data
Forsman, et al. [18]	Mixed species level data
Fransson and Weber [19]	Season (migration) had insufficient independent studies for inclusion (K = 2)
Ghalambor and Martin [20]	Mixed species level data
Gomez-Serrano [21]	Experimental portion of study used humans as "predator" treatment
Griesser [22]	for experiments 1: treatment and control observations cannot be compared (different observation durations), for experiment 2: testing information content of calls
Griesser [23]	No control
Groenewoud, et al. [24]	No predator treatment
Holthuijzen [25]	No relevant treatment, assessing if birds recognize heterospecific alarm calls
Hua, et al. [26]	Mixed species level data
Huang, et al. [27]	Test of heterospecific information use
Hunts, et al. [28]	Mixed species level data
Ibanez-Alamo, et al. [29]	Response type (fecal sac removals) not replicated in any other study. Also, no clear predicted direction of effect based on theory.
Iglesias, et al. [30]	No control
Jones and Sieving [31]	Species level data cannot be extracted for control/treatment contrasts

Journey, et al. [32]	No s.e. (standard error) provided for species level data
Keen, et al. [33]	Social learning of novel cue
Kerman, et al. [34]	No control
Leavesley and Magrath [35]	No treatment: study tests whether trills convey predator information
Macleod, et al. [36]	Response variable is cumulative mass gain expressed as percentage (i.e., control and treatment scaled to same range from 0 to 100)
Madden, et al. [37]	mobbing calls used for treatment were generated in response to humans
Martinez, et al. [38]	Could not extract species level data- figure resolution too low to extract overlapping data points
McIntyre, et al. [39]	Effect direction not extractable
Morosinotto, et al. [40]	response variable = testosterone excluded because no clear predicted effect (authors themselves stated no single prediction)
Nilsson and Nord [41]	Not relevant - no predator treatment
Nocera and Ratcliffe [42]	Mixed species level data
Pascual and Senar [43]	Manipulation is distance to cover
Poysa, et al. [44]	Not relevant - no predator treatment
Rajala, et al. [45]	No control
Rands and Cuthill [46]	manipulation is human threat
Roncalli, et al. [47]	Response variables not relevant (egg touches, egg rejection)
Schneider and Griesser [48]	Cannot extract behavioural response to treatments in isolation
Serra and Fernandez [49]	manipulation is human threat
Sieving, et al. [50]	Response variables is structure of acoustic response -species specific, not generalizable
Thompson, et al. [51]	Not relevant - no predator treatment
Tilgar and Moks [52]	Mixed species level data
Tilgar, et al. [53]	manipulation is human threat
Tolvanen, et al. [54]	Not relevant - no predator treatment
Turney and Godin [55]	Mixed species level data
Tvardikova and Fuchs [56]	Mixed species level data
Williams and Lindell [57]	Not relevant - no predator treatment
Williamson and Fagan [58]	Mixed species level data
Zanette, et al. [59]	Response subcategory (cFOS levels) not used in any other study, also, no clear directional prediction

Table S4: List of variables extracted from articles included in the meta-analysis and rationale.

**Variable**	**Rationale**
Publication year	Assessment of publication bias via time lag effect
Focal species	To allow us to control for phylogeny in meta-regression
Predator (species name if single species, else "multi-species")	Collected to provide more complete meta-data for review and cross checking "predator guild" variable.
Predator guild (bird, mammal, fish, reptile, not specified, or multiple guilds)	To allow exploratory analyses of whether specific predator guilds elicit stronger responses than others.
Adult or nest predator	To allow exploratory analyses of whether magnitude of response differs in response to adult versus nest/nestling predators.
Setting of the study (field, lab, semi-natural)	To allow exploratory analyses of whether magnitude of response differs as a function of study setting. If differences exist, this would have important implications for whether laboratory studies can be extrapolated to field conditions.
Treatment (A, O, V, or combination thereof)	Key variables of interest to test: 1) whether different modalities of information elicit different response magnitudes consistent with assumption that different modalities provide different degrees of certainty about predation risk, and 2) to evaluate how response to multi-modal cues differ from responses to unimodal cues. Detailed rationale provided in introduction.
Season (breeding, non-breeding)	To allow exploratory analyses of effect of season (breeding versus non-breeding). For example, parents may value personal survival differently when
Type of comparison (within- or among-subject)	To allow exploratory analyses of whether estimated effect sizes differ for within- versus among-subject designs based on the fact that within-subject designs are generally more powerful because they control for among-subject variability.
Treatment duration (days)	To allow exploratory analyses of effect of treatment duration. Longer treatments may elicit stronger responses for traits that are plastic over longer time scales (e.g., life history traits), but may elicit smaller response for traits that are highly plastic if longer exposure leads to habituation.
Control type (blank, disturbance, non-predator)	To allow for exploratory analyses of whether control type effects estimated response to manipulations of perceived predation risk. Blank controls compare unmanipulated birds to birds following predator manipulations -and thus do not control for disturbance associated with conducting a treatment. "Disturbance" controls employ a matched disturbance (e.g., time that observer is present, presence of novel objects), or "non-predator" controls control for both non-biological and biological
Sex of focal individuals (male, female, both)	To allow exploratory analyses of sex-related differences in response magnitude.
Age of focal individuals (A = Adults, N = Nestlings, J = Juveniles, E = Eggs)	To allow exploratory analyses of age-related differences in response magnitude.

Supplementary References

1 O'Dea, R. E. *et al.* Preferred reporting items for systematic reviews and meta-analyses in ecology and evolutionary biology: a PRISMA extension. *Biol. Rev. Camb. Philos. Soc.* **96**, 1695-1722, doi:10.1111/brv.12721 (2021).

2 Amo, L., Caro, S. P. & Visser, M. E. Sleeping birds do not respond to predator odour. *Plos One* **6**, doi:10.1371/journal.pone.0027576 (2011).

3 Amo, L., Visser, M. E. & van Oers, K. Smelling out predators is innate in birds. *Ardea* **99**, 177-184, doi:10.5253/078.099.0207 (2011).

4 Andreasson, F., Nord, A. & Nilsson, J. A. Age-dependent effects of predation risk on night-time hypothermia in two wintering passerine species. *Oecologia* **189**, 329-337, doi:10.1007/s00442-018-04331-7 (2019).

5 Antze, B. & Koper, N. Noisy anthropogenic infrastructure interferes with alarm responses in Savannah sparrows (*Passerculus sandwichensis*). *R. Soc. Open Sci.* **5**, doi:10.1098/rsos.172168 (2018).

6 Atkins, A., Redpath, S. M., Little, R. M. & Amar, A. Experimentally manipulating the landscape of fear to manage problem animals. *J. Wildl. Manag.* **81**, 610-616, doi:10.1002/jwmg.21227 (2017).

7 Aviles, J. M., Parejo, D. & Exposito-Granados, M. Avian and rodent responses to the olfactory landscape in a Mediterranean cavity community. *Oecologia* **191**, 73-81, doi:10.1007/s00442-019-04487-w (2019).

8 Berziņš, A. *et al.* Mobbing as a trade-off between safety and reproduction in a songbird. *Behav. Ecol.* **21**, 1054-1060, doi:10.1093/beheco/arq104 (2010).

9 Blackwell, B. F., Seamans, T. W., Pfeiffer, M. B. & Buckingham, B. N. European Starling (*Sturnus vulgaris*) reproduction undeterred by predator scent inside nest boxes. *Can. J. Zool.* **96**, 980-986, doi:10.1139/cjz-2017-0299 (2018).

10 Breviglieri, C. P. B. & Romero, G. Q. Snakes and forbidden fruits: non-consumptive effects of snakes on the behaviors of frugivorous birds. *Behav. Ecol. Sociobiol.* **70**, 777-783, doi:10.1007/s00265-016-2101-7 (2016).

11 Cimprich, D. A., Woodrey, M. S. & Moore, F. R. Passerine migrants respond to variation in predation risk during stopover. *Anim. Behav.* **69**, 1173-1179, doi:10.1016/j.anbehav.2004.07.021 (2005).

12 Coslovsky, M. & Richner, H. Increased predation risk on mothers affects survival of parasites feeding on the offspring. *Anim. Behav.* **81**, 1071-1075, doi:10.1016/j.anbehav.2011.02.023 (2011).

13 da Cunha, F. C. R., Fontenelle, J. C. R. & Griesser, M. The presence of conspecific females influences male-mobbing behavior. *Behav. Ecol. Sociobiol.* **71**, doi:10.1007/s00265-017-2267-7 (2017).

14 Davies, N. B. & Welbergen, J. A. Cuckoo-hawk mimicry? An experimental test. *Proceedings of the Royal Society B-Biological Sciences* **275**, 1817-1822, doi:10.1098/rspb.2008.0331 (2008).

15 Dutour, M., Lena, J. P. & Lengagne, T. Mobbing behaviour varies according to predator dangerousness and occurrence. *Anim. Behav.* **119**, 119-124, doi:10.1016/j.anbehav.2016.06.024 (2016).

16 Fardell, L. L., Nano, C. E. M., Pavey, C. R. & Dickman, C. R. Small prey animal habitat use in landscapes of fear: Effects of predator presence and human activity along an urban disturbance gradient. *Front. Ecol. Evol.* **9**, doi:10.3389/fevo.2021.750094 (2021).

17 Forsman, J. T. & Monkkonen, M. Responses by breeding birds to heterospecific song and mobbing call playbacks under varying predation risk. *Anim. Behav.* **62**, 1067-1073, doi:10.1006/anbe.2001.1856 (2001).

18 Forsman, J. T., Monkkonen, M., Inkeroinen, J. & Reunanen, P. Aggregate dispersion of birds after encountering a predator: experimental evidence. *J. Avian Biol.* **29**, 44-48, doi:10.2307/3677339 (1998).

19 Fransson, T. & Weber, T. P. Migratory fuelling in blackcaps (*Sylvia atricapilla*) under perceived risk of predation. *Behav. Ecol. Sociobiol.* **41**, 75-80, doi:10.1007/s002650050366 (1997).

20 Ghalambor, C. K. & Martin, T. E. Fecundity-survival trade-offs and parental risk-taking in birds. *Science* **292**, 494-497, doi:10.1126/science.1059379 (2001).

21 Gomez-Serrano, M. A. Four-legged foes: dogs disturb nesting plovers more than people do on tourist beaches. *Ibis* **163**, 338-352, doi:10.1111/ibi.12879 (2021).

22 Griesser, M. Referential calls signal predator behavior in a group-living bird species. *Current Biology* **18**, 69-73, doi:10.1016/j.cub.2007.11.069 (2008).

23 Griesser, M. Do warning calls boost survival of signal recipients? Evidence from a field experiment in a group-living bird species. *Frontiers in Zoology* **10**, doi:10.1186/1742-9994-10-49 (2013).

24 Groenewoud, F., Kingma, S. A., Bebbington, K., Richardson, D. S. & Komdeur, J. Experimentally induced antipredator responses are mediated by social and environmental factors. *Behav. Ecol.* **30**, 986-992, doi:10.1093/beheco/arz039 (2019).

25 Holthuijzen, W. A. Stranger danger: Acoustic response of the Veery (Catharus fuscescens) via heterospecific eavesdropping on the Tufted Titmouse (*Baeolophus bicolor*). *Wilson Journal of Ornithology* **130**, 168-179, doi:10.1676/16-149.1 (2018).

26 Hua, F. Y., Fletcher, R. J., Sieving, K. E. & Dorazio, R. M. Too risky to settle: avian community structure changes in response to perceived predation risk on adults and offspring. *Proceedings of the Royal Society B-Biological Sciences* **280**, doi:10.1098/rspb.2013.0762 (2013).

27 Huang, P., Sieving, K. E. & St Mary, C. M. Heterospecific information about predation risk influences exploratory behavior. *Behav. Ecol.* **23**, 463-472, doi:10.1093/beheco/arr212 (2012).

28 Hunts, C., Heather, M., Martinez, A. E. & Parra, E. Experimental evidence of alarm eavesdropping on Amazonian mixed-flock followers by two social sentinel species: the dusky-throated antshrike (*Thamnomanes ardesicus*) and the cinereous antshrike (*T. schistogynus*). *Ornitol. Neotrop.* **30**, 224-228 (2019).

29 Ibanez-Alamo, J. D., Sanllorente, O., Arco, L. & Soler, M. Does nest predation risk induce parent birds to eat nestlings' fecal sacs? An experimental study. *Ann. Zool. Fenn.* **50**, 71-78, doi:10.5735/086.050.0106 (2013).

30 Iglesias, T. L., Stetkevitch, R. C. & Patricelli, G. L. Dead heterospecifics as cues of risk in the environment: Does size affect response? *Behaviour* **151**, 1-22, doi:10.1163/1568539x-00003120 (2014).

31 Jones, H. H. & Sieving, K. E. Foraging ecology drives social information reliance in an avian eavesdropping community. *Ecol. Evol.* **9**, 11584-11597, doi:10.1002/ece3.5561 (2019).

32 Journey, L., Drury, J. P., Haymer, M., Rose, K. & Blumstein, D. T. Vivid birds respond more to acoustic signals of predators. *Behav. Ecol. Sociobiol.* **67**, 1285-1293, doi:10.1007/s00265-013-1556-z (2013).

33 Keen, S. C., Cole, E. F., Sheehan, M. J. & Sheldon, B. C. Social learning of acoustic anti-predator cues occurs between wild bird species. *Proceedings of the Royal Society B-Biological Sciences* **287**, doi:10.1098/rspb.2019.2513 (2020).

34 Kerman, K., Sieving, K. E., St Mary, C. & Avery, M. L. Social conformity affects experimental measurement of boldness in male but not female monk parakeets (*Myiopsitta monachus*). *Behaviour* **155**, 1025-1050, doi:10.1163/1568539x-00003519 (2018).
35 Leavesley, A. J. & Magrath, R. D. Communicating about danger: urgency alarm calling in a bird. *Anim. Behav.* **70**, 365-373, doi:10.1016/j.anbehav.2004.10.017 (2005).
36 Macleod, R., Gosler, A. G. & Cresswell, W. Diurnal mass gain strategies and perceived predation risk in the great tit *Parus major*. *J. Anim. Ecol.* **74**, 956-964, doi:10.1111/j.1365-2656.2005.00993.x (2005).
37 Madden, J. R., Kilner, R. M. & Davies, N. B. Nestling responses to adult food and alarm calls: 1. Species-specific responses in two cowbird hosts. *Anim. Behav.* **70**, 619-627, doi:10.1016/j.anbehav.2004.11.019 (2005).
38 Martinez, A. E., Parra, E., Collado, L. F. & Vredenburg, V. T. Deconstructing the landscape of fear in stable multi-species societies. *Ecology* **98**, 2447-2455, doi:10.1002/ecy.1935 (2017).
39 McIntyre, E., Horn, A. G. & Leonard, M. L. Do nestling Tree Swallows (*Tachycineta bicolor*) respond to parental alarm calls? *Auk* **131**, 314-320, doi:10.1642/auk-13-235.1 (2014).
40 Morosinotto, C. *et al.* Maternal transfer of androgens in eggs is affected by food supplementation but not by predation risk. *J. Avian Biol.* **47**, 629-641, doi:10.1111/jav.00874 (2016).
41 Nilsson, J. A. & Nord, A. The use of the nest for parental roosting and thermal consequences of the nest for nestlings and parents. *Behav. Ecol. Sociobiol.* **71**, doi:10.1007/s00265-017-2400-7 (2017).
42 Nocera, J. J. & Ratcliffe, L. M. Migrant and resident birds adjust antipredator behavior in response to social information accuracy. *Behav. Ecol.* **21**, 121-128, doi:10.1093/beheco/arp161 (2010).
43 Pascual, J. & Senar, J. C. Differential effects of predation risk and competition over vigilance variables and feeding success in Eurasian siskins (*Carduelis spinus*). *Behaviour* **150**, 1665-1687, doi:10.1163/1568539x-00003114 (2013).
44 Poysa, H., Ruusila, V., Milonoff, M. & Virtanen, J. Ability to assess nest predation risk in secondary hole-nesting birds: an experimental study. *Oecologia* **126**, 201-207, doi:10.1007/s004420000512 (2001).
45 Rajala, M., Ratti, O. & Suhonen, J. Age differences in the response of willow tits (*Parus montanus*) to conspecific alarm calls. *Ethology* **109**, 501-509, doi:10.1046/j.1439-0310.2003.00890.x (2003).
46 Rands, S. A. & Cuthill, I. C. Separating the effects of predation risk and interrupted foraging upon mass changes in the blue tit *Parus caeruleus*. *Proceedings of the Royal Society B-Biological Sciences* **268**, 1783-1790, doi:10.1098/rspb.2001.1653 (2001).
47 Roncalli, G., Soler, M., Ruiz-Raya, F., Serrano-Martin, A. J. & Ibanez-Alamo, J. D. Predation risk affects egg-ejection but not recognition in blackbirds. *Behav. Ecol. Sociobiol.* **73**, doi:10.1007/s00265-019-2668-x (2019).
48 Schneider, N. A. & Griesser, M. The alarm call system of breeding Brown Thornbills (*Acanthiza pusilla*): self-defence or nest defence? *J. Ornithol.* **155**, 987-996, doi:10.1007/s10336-014-1085-1 (2014).
49 Serra, C. & Fernandez, G. J. Reduction of nestlings' vocalizations in response to parental alarm calls in the Southern house wren, *Troglodytes musculus*. *J. Ornithol.* **152**, 331-336, doi:10.1007/s10336-010-0595-8 (2011).
50 Sieving, K. E., Hetrick, S. A. & Avery, M. L. The versatility of graded acoustic measures in classification of predation threats by the tufted titmouse *Baeolophus bicolor*: exploring a mixed

framework for threat communication. *Oikos* **119**, 264-276, doi:10.1111/j.1600-0706.2009.17682.x (2010).
51 Thompson, A. M. *et al.* The influence of fledgling location on adult provisioning: a test of the blackmail hypothesis. *Proceedings of the Royal Society B-Biological Sciences* **280**, doi:10.1098/rspb.2013.0558 (2013).
52 Tilgar, V. & Moks, K. Increased risk of predation increases mobbing intensity in tropical birds of French Guiana. *J. Trop. Ecol.* **31**, 243-250, doi:10.1017/s0266467415000061 (2015).
53 Tilgar, V., Saag, P., Kulavee, R. & Mand, R. Behavioral and physiological responses of nestling pied flycatchers to acoustic stress. *Horm. Behav.* **57**, 481-487, doi:10.1016/j.yhbeh.2010.02.006 (2010).
54 Tolvanen, J. *et al.* Interspecific information on predation risk affects nest site choice in a passerine bird. *BMC Evol. Biol.* **18**, doi:10.1186/s12862-018-1301-3 (2018).
55 Turney, S. & Godin, J. G. J. To forage or hide? Threat-sensitive foraging behaviour in wild, non-reproductive passerine birds. *Current Zoology* **60**, 719-728, doi:10.1093/czoolo/60.6.719 (2014).
56 Tvardikova, K. & Fuchs, R. Do birds behave according to dynamic risk assessment theory? A feeder experiment. *Behav. Ecol. Sociobiol.* **65**, 727-733, doi:10.1007/s00265-010-1075-0 (2011).
57 Williams, S. M. & Lindell, C. A. Nuclear species in Peruvian Amazonian mixed-species flocks are differentially attractive to transient species and to each other. *Wilson Journal of Ornithology* **130**, 131-139 (2018).
58 Williamson, J. L. & Fagan, M. E. Predator playback, foraging height, and phylogeny affect gap crossing behavior in tropical forest birds. *Ornitol. Neotrop.* **28**, 163-174 (2017).
59 Zanette, L. Y., Hobbs, E. C., Witterick, L. E., MacDougall-Shackleton, S. A. & Clinchy, M. Predator-induced fear causes PTSD-like changes in the brains and behaviour of wild animals. *Scientific Reports* **9**, doi:10.1038/s41598-019-47684-6 (2019).
