# Supplementary material for: A systematic review and meta-analysis of unimodal and multimodal predation risk assessment in birds
Source: Nat Commun. 2024 May 18;15:4240. doi: 10.1038/s41467-024-48702-6 (PMC11102462; doi:10.1038/s41467-024-48702-6)
Supplement: Supplementary file 3 — Reporting Summary [file 41467_2024_48702_MOESM3_ESM.pdf]

nature portfolio

Corresponding author(s): Kimberley J. Mathot

Last updated by author(s): Apr 6, 2024

Reporting Summary

Nature Portfolio wishes to improve the reproducibility of the work that we publish. This form provides structure for consistency and transparency in reporting. For further information on Nature Portfolio policies, see our Editorial Policies and the Editorial Policy Checklist.

Statistics

For all statistical analyses, confirm that the following items are present in the figure legend, table legend, main text, or Methods section.

n/a	Confirmed	
[ ]	[x]	The exact sample size (*n*) for each experimental group/condition, given as a discrete number and unit of measurement
[ ]	[x]	A statement on whether measurements were taken from distinct samples or whether the same sample was measured repeatedly
[ ]	[x]	The statistical test(s) used AND whether they are one- or two-sided<br>*Only common tests should be described solely by name; describe more complex techniques in the Methods section.*
[ ]	[x]	A description of all covariates tested
[ ]	[x]	A description of any assumptions or corrections, such as tests of normality and adjustment for multiple comparisons
[ ]	[x]	A full description of the statistical parameters including central tendency (e.g. means) or other basic estimates (e.g. regression coefficient) AND variation (e.g. standard deviation) or associated estimates of uncertainty (e.g. confidence intervals)
[ ]	[x]	For null hypothesis testing, the test statistic (e.g. *F*, *t*, *r*) with confidence intervals, effect sizes, degrees of freedom and *P* value noted<br>*Give P values as exact values whenever suitable.*
[x]	[ ]	For Bayesian analysis, information on the choice of priors and Markov chain Monte Carlo settings
[ ]	[x]	For hierarchical and complex designs, identification of the appropriate level for tests and full reporting of outcomes
[ ]	[x]	Estimates of effect sizes (e.g. Cohen's *d*, Pearson's *r*), indicating how they were calculated

Our web collection on statistics for biologists contains articles on many of the points above.

Software and code

Policy information about availability of computer code

Data collection	Studies were identified using the online databases Web of Science and Scopus. Study abstracts were screened using Rayyan. WebPlotDigitizer v4.1 was used to obtain data from figures. We obtained the avian phylogenetic tree from Jetz et al. 2012 (Nature 491: 444-448). All data collected from each identified study can be accessed from the archived project on OSF using the following link: https://doi.org/10.17605/OSF.IO/9VMZX
Data analysis	The RStudio interface was used to perform all analyses in R v4.3.1. The following packages (with version numbers) were used to run analyses: grid, parallel, stats, graphics, grDevices, utils, datasets, methods and base, magick(v.2.7.5), raster(v.3.6-23), sp(v.2.0-0), png(v.0.1-8), cowplot (v.1.1.1), ggthemes(v.4.2.4), maps(v.3.4.1), readxl(v.1.4.3), ggstance(v.0.3.6), ggtree(v.3.8.2), miWQS(v.0.4.4), pander(v.0.6.5), kableExtra (v.1.3.4), MuMIn(v.1.47.5), emmeans(v.1.8.8), clubSandwich(v.0.5.10), ape(v.5.7-1), ggalluvial(v.0.12.5), alluvial(v.0.1-2), patchwork(v.1.1.3), metafor(v.4.2-0), numDeriv(v.2016.8-1.1), metadat(v.1.2-0), Matrix(v.1.6-1), orchaRd(v.2.0), here(v.1.0.1), lubridate(v.1.9.2), forcats(v.1.0.0), stringr(v.1.5.0), dplyr(v.1.1.3), purrr(v.1.0.2), readr(v.2.1.4), tidyr(v.1.3.0), tibble(v.3.2.1), ggplot2(v.3.4.4), tidyverse(v.2.0.0) and pacman (v.0.5.1), mathjaxr(v.1.6-0), rstudioapi(v.0.15.0), jsonlite(v.1.8.8), magrittr(v.2.0.3), ggbeeswarm(v.0.7.2), TH.data(v.1.1-2), estimability (v.1.4.1), farver(v.2.1.1), rmarkdown(v.2.24), MCMCpack(v.1.6-3), vctrs(v.0.6.5), memoise(v.2.0.1), terra(v.1.7-39), base64enc(v.0.1-3), webshot(v.0.5.5), htmltools(v.0.5.7), truncnorm(v.1.0-9), cellranger(v.1.1.0), Formula(v.1.2-5), gridGraphics(v.0.5-1), glm2(v.1.2.1), htmlwidgets(v.1.6.2), matrixNormal(v.0.1.1), sandwich(v.3.0-2), zoo(v.1.8-12), cachem(v.1.0.8), lifecycle(v.1.0.4), pkgconfig(v.2.0.3), R6 (v.2.5.1), fastmap(v.1.1.1), digest(v.0.6.34), aplot(v.0.2.0), colorspace(v.2.1-0), rprojroot(v.2.0.3), Hmisc(v.5.1-0), labeling(v.0.4.3), invgamma (v.1.1), latex2exp(v.0.9.6), fansi(v.1.0.4), timechange(v.0.2.0), mgcv(v.1.9-0), httr(v.1.4.7), compiler(v.4.3.1), withr(v.2.5.2), htmlTable(v.2.4.1), backports(v.1.4.1), condMVNorm(v.2020.1), highr(v.0.10), MASS(v.7.3-60), quantreg(v.5.97), tools(v.4.3.1), vipor(v.0.4.5), foreign(v.0.8-84), beeswarm(v.0.4.0), nnet(v.7.3-19), glue(v.1.7.0), nlme(v.3.1-163), checkmate(v.2.2.0), cluster(v.2.1.4), generics(v.0.1.3), gtable(v.0.3.4), tzdb (v.0.4.0), data.table(v.1.14.8), hms(v.1.1.3), xml2(v.1.3.5), utf8(v.1.2.3), pillar(v.1.9.0), yulab.utils(v.0.0.9), splines(v.4.3.1), gmm(v.1.8), treeio (v.1.24.3), lattice(v.0.21-8), survival(v.3.5-7), SparseM(v.1.81), tidyselect(v.1.2.0), knitr(v.1.43), gridExtra(v.2.3), svglite(v.2.1.1), mcmc(v.0.9-7),

stats4(v.4.3.1), xfun(v.0.40), Rsolnp(v.1.16), tmvmixnorm(v.1.1.1), stringi(v.1.7.12), lazyeval(v.0.2.2), ggfun(v.0.1.2), yaml(v.2.3.8), evaluate (v.0.21), codetools(v.0.2-19), ggplotify(v.0.1.2), cli(v.3.6.2), rpart(v.4.1.19), xtable(v.1.8-4), systemfonts(v.1.0.4), munsell(v.0.5.0), Rcpp (v.1.0.12), coda(v.0.19-4), MatrixModels(v.0.5-2), viridisLite(v.0.4.2), mvtnorm(v.1.2-3), tidytree(v.0.4.5), rlist(v.0.4.6.2), scales(v.1.2.1), tmvtnorm(v.1.5), rlang(v.1.1.3), rvest(v.1.0.3) and multcomp(v.1.4-25).
All code required to reproduce the analyses and figures presented in the manuscript can be accessed from the archived project on OSF using the following link: https://doi.org/10.17605/OSF.IO/9VMZX

For manuscripts utilizing custom algorithms or software that are central to the research but not yet described in published literature, software must be made available to editors and reviewers. We strongly encourage code deposition in a community repository (e.g. GitHub). See the Nature Portfolio guidelines for submitting code & software for further information.

Data

Policy information about availability of data

All manuscripts must include a data availability statement. This statement should provide the following information, where applicable:

- Accession codes, unique identifiers, or web links for publicly available datasets
- A description of any restrictions on data availability
- For clinical datasets or third party data, please ensure that the statement adheres to our policy

All data used in the present study can are archived on OSF and can be found at https://doi.org/10.17605/OSF.IO/9VMZX
All data are available for re-use under the following licence: CC-By Attribution 4.0 International. The data are available for the main analysis is provided in the file “dat_19_07_2023_spp.csv”. Descriptions of all meta-data are provided in “Meta-data.csv”, and data related to study species and study country required to generate figures is provided is “Species list.xlsx” and “StudyCountries.xlsx”, respectively.

Research involving human participants, their data, or biological material

Policy information about studies with human participants or human data. See also policy information about sex, gender (identity/presentation), and sexual orientation and race, ethnicity and racism.

Reporting on sex and gender	NA
Reporting on race, ethnicity, or other socially relevant groupings	NA
Population characteristics	NA
Recruitment	NA
Ethics oversight	NA

Note that full information on the approval of the study protocol must also be provided in the manuscript.

Field-specific reporting

Please select the one below that is the best fit for your research. If you are not sure, read the appropriate sections before making your selection.

[x] Life sciences [ ] Behavioural & social sciences [ ] Ecological, evolutionary & environmental sciences

For a reference copy of the document with all sections, see nature.com/documents/nr-reporting-summary-flat.pdf

Life sciences study design

All studies must disclose on these points even when the disclosure is negative.

Sample size	Our research sample includes 645 estimates extracted from 116 studies representing 87 species. Sample sizes were based on the number of relevant studies we were able to include in our meta-analysis based on the data exclusions outlined below, and the number of estimates that could be extracted from each of those studies.
Data exclusions	For a study to be included in our meta-analysis, it had to fulfill 5 criteria. 1) The study had to present an experimental manipulation of perceived predation risk. These had to experimentally provide cues of predator presence (olfactory, visual, or auditory). For olfactoryc cues, we considered both presentation of chemicals obtained directly from predators as well as synthetic predator odours. For acoustic cues, we only included vocalizations made by known predators or mobbing/alarm calls made by the focal species. We did not include studies that aimed to test whether a cue was recognized by birds (e.g., presentation of a novel predator). We did not consider the presence of human observers alone as an experimental manipulation of perceived predation. Similarly, we did not consider mobbing or alarm calls produced in response to humans as a relevant manipulation of perceived predation risk. We included studies that manipulated perceived predation risk using live predators as long as the presence/absence of the predator was determined experimentally (e.g., caged predator, or presented via falconer). Studies that manipulated predation risk without providing cues related to the presence of actual predators were not included. For example, we excluded studies that manipulated the size of the nest box entrance so that some were accessible by predators and others were not, or studies that manipulated landscape features (e.g., distance to obstructive cover, distance to protective cover) that alter the ability to detect and/or evade predators. We also did not include studies that manipulated predation risk using predator removals or exclusions, as these did not report the predator cues (type, frequency) that were encountered in the control groups (i.e., non-removal plots or outside

exclusions).
2) The study had to provide data on behaviour, life history, or physiology/morphology as a function of manipulated perceived risk. The full list of traits included in the meta-analysis and their definitions is provided in Supplementary Information Table S2.
3) The study had to allow for the calculation of effect size for a behavioural, life history, or physiological variable in response to a manipulation of perceived predation risk as described in (1). The study had to include a control for the manipulation, such as data on the response variable prior to the experiment in the same set of individuals (Before-After-Control-Impact (BACI) or within-subject design), or contrasts between sets of individuals exposed to the manipulation and individual not exposed to the manipulation (among-subject design). Studies that only contrasted different manipulations of perceived risk (e.g., response to visual cue versus response to acoustic cue) were not included. We excluded any estimates for which there were less than N = 3 individuals in a given treatment group because the standard deviation (SD) could not be estimated well with small sample sizes (see below calculation details).
4) The study had to be conducted on birds and present species-specific results. Studies that presented mixed-species responses (e.g., the average response of a mixed-species flock) were not included in the meta-analysis.
5) We initially considered any behavioural, life history, or physiological trait if the study fulfilled the four criteria listed above. However, following full-text screening of all articles, we removed studies/estimates if there were not at least N = 3 studies that provided extractable data for that response variable.

Replication	NA - we were extracting estimates from already published studies.
Randomization	NA - we were extracting estimates from already published studies.
Blinding	NA - we were extracting estimates from already published studies.

Reporting for specific materials, systems and methods

We require information from authors about some types of materials, experimental systems and methods used in many studies. Here, indicate whether each material, system or method listed is relevant to your study. If you are not sure if a list item applies to your research, read the appropriate section before selecting a response.

Materials & experimental systems

n/a	Involved in the study	
☒	☐	Antibodies
☒	☐	Eukaryotic cell lines
☒	☐	Palaeontology and archaeology
☒	☐	Animals and other organisms
☒	☐	Clinical data
☒	☐	Dual use research of concern
☒	☐	Plants

Methods

n/a	Involved in the study	
☒	☐	ChIP-seq
☒	☐	Flow cytometry
☒	☐	MRI-based neuroimaging

Plants

Seed stocks	NA
Novel plant genotypes	NA
Authentication	NA
